# Quinoline Alkaloids Isolated from *Choisya* Aztec-Pearl and Their Contribution to the Overall Antinociceptive Activity of This Plant

**DOI:** 10.1371/journal.pone.0164998

**Published:** 2016-10-21

**Authors:** Patricia Ribeiro de Carvalho, Denise Ricoy Ropero, Mariana Martins Pinheiro, Patricia Dias Fernandes, Fabio Boylan

**Affiliations:** 1 Universidade Federal do Rio de Janeiro, Instituto de Ciências Biomédicas, Laboratório de Farmacologia da Dor e da Inflamação, Rio de Janeiro, Brazil; 2 School of Pharmacy and Pharmaceutical Sciences, Trinity Biomedical Sciences Institute, Trinity College Dublin, Dublin, Ireland; Institute of medical research and medicinal plant studies, CAMEROON

## Abstract

*Choisya* ‘Aztec-Pearl’, a hybrid of *Choisya ternata* and *Choisya dumosa* var. *arizonica*, had the antinociceptive activity in the ethanol extract (EECA) of its leaves evaluated. Two quinoline alkaloids, anhydroevoxine (A) and choisyine (C), isolated from these leaves were also tested. The results obtained pointed out to a very high antinociceptive activity measured by the hot plate model for EECA (at doses of 10, 30 and 100 mg/kg) as well as for A and C (at doses of 1, 3 and 10 mg/kg). The magnitude of the activity was two-fold higher than the one observed for the morphine treated animals for the higher doses of extracts/compounds (30, 100 mg/kg and 3, 10 mg/kg respectively). The mechanism of action for this activity was also investigated and it seems that for EECA as well as A and C, the opiate system plays an important role. Results have also shown that the nitric oxide (NO) system also play a pivotal role in the case of EECA and A while for C it seems that the cholinergic system have some involvement. The acute toxicity was evaluated for EECA with results showing no important toxic effect.

## Introduction

*Choisya* ‘Aztec-Pearl’ is a hybrid of *Choisya ternata* and *Choisya dumosa* var. *arizonica*. These two species were artificially crossed in 1982 [1]. This hybrid is an intermediate between its parents; it is less robust in stature than *C*. *ternata* and it produces larger flowers.

*Choisya ternata* Kunth, known as Mexican orange or Mexican orange blossom, is a widely cultivated species that was first introduced into Britain from Mexico in 1825 [2,3]. *C*. *dumosa* var. *arizonica* is also commonly known as mexican-orange or starleaf and to Mexicans as sorilla or zorillo. Both species have been attributed to contain toxic compounds [4] that could potentially cause harm to the livestock where they grow but the presence of such compounds in the plant has not yet been proved.

Plants from the Rutaceae family are widely used in folk medicine. Different species are used as tonic, febrifuge, against inflammation and microbial infections and for the treatment of malaria [5–9]. A literature review on the chemical constituents of different species found within this family has shown the occurrence of quinoline alkaloids [10–13].

Previous studies by our group have shown that the extracts obtained from *C*. *ternata*, as well as the compounds present in its essential oil possessed antinociceptive activity [14, 15]. This lead to the evaluation of the non-volatile chemical composition of this hybrid as well as to the investigation of a potential pain relief action in the crude ethanol extract and its isolated compounds.

The international association for the study of pain defines nociceptive pain as a pain that arises from actual or threatened damage to non-neural tissue and due to the activation of nociceptors. This term is designed to contrast with neuropathic pain. The term is used to describe pain occurring with a normally functioning somatosensory nervous system to contrast with the abnormal function seen in neuropathic pain [16]. The importance of pain resides in the fact that it is the major symptom of several different illnesses. It can also impair mobility causing a lower quality of life in the patients who are suffering from it [17]. There are several pharmacologically and biochemically described modulations of pain involving an array of different cell types and mediators [18]. Although there are protocols for the management of peripheral and central nervous system pain, the potential of side effects developing are of major concern such as gastric problems in the case of the non-steroidal anti-inflammatory drugs (NSAID) for the relief of peripheral pain. There is also the possibility of addiction in the case of the opiates for the relief of central nervous system pain [19].

In this way the discovery of new compounds with antinociceptive activity is really important therapeutically for the treatment of pain, especially in the research field of medicinal plants and natural products. Ethnopharmacology plays an important role in the research of renowned species providing new chemical entities of utmost importance [20]. Our research group has contributed to this field in many ways such as validating medicinal plants used popularly for the management of pain and discovering of new antinociceptive molecules from medicinal plants [21–30].

## Material and Methods

### General

Melting points were recorded in oC on a SMP-1 (Stuart Scientific, UK). Optical rotations [α] were measured in a 1 dm. tube using an Alltech AA-55 polarimeter for Optical Activity Ltd. EI mass spectra were measured on Waters Micromass GCT Premier Mass spectrometer and Mass Lynx V4.1 software. The 1H and 13C NMR spectra were recorded on a BRUKER TOPSPIN 2.1 NMR System 400 and 600 MHz spectrometers, using standard Bruker pulse sequences, as well as both uni- and bi-dimensional technique NMR spectra (1H-1H COSY, HMBC, HMQC).

Column chromatography was performed using silica gel 60 (70–230 mesh; Merck). Thin layer chromatography (TLC), aluminium sheets of silica gel 60 F254, (230–400 Mesh ASTM, 20 cm × 20 cm, layer thickness 0.25mm, Merck) were used for analytical purposes and the compounds were visualized under ultraviolet light. Solvents used for extraction and chromatography were of analytical grade—with an exception to ethanol, which was of commercial grade.

### Plant Material

Fresh leaves from one specimen of *C*. Aztec-Pearl were collected in Dublin, Ireland in September 2013. The plant was collected on a private garden owned by Professor Trevor Hodkinson, Trinity College Dublin, who gave permission for the collection of its leaves. Prof. Hodkinson also identified the species and a voucher specimen (ref. TCD Hodkinson & Ropero 01) was deposited in the Herbarium of Trinity College, Dublin. It is important to mention that the studied plant is not either an endangered or protected species.

### Extraction of *Choisya* Aztec-Pearl

The extraction of leaves (850g) of *C*. Aztec-Pearl was performed using Soxhlet apparatus for 72 hours. The solvent used in this extraction was ethanol. The crude ethanol extract of leaves (EECA) was reduced to near dryness under reduced pressure using a rotary evaporator in a heating bath (187.3 g). EECA was dissolved in 20% ethanol and 80% water. Liquid-liquid extractions were performed with the following solvents: hexane, dichloromethane, ethyl acetate and *n*-butanol, yielding 19g hexane; 16g dichloromethane; 21g ethyl acetate; 28g butanol extracts, respectively.

### Analysis of *Choisya* Aztec-Pearl Extracts

The analysis of the dichloromethane extracts (DCME) of the leaves showed a richness in alkaloids. Fifteen grams of the DCME from leaves were subjected to a chromatography column (25cm) over silica gel (150 g, 70–230 Mesh, Merck) eluted with gradients of hexane: ethyl acetate: methanol to yield 157 fractions, which were grouped according to their similarity, allowing a total of 20 junctions. Junction 11 was washed with methanol and the white crystals formed were identified as anhydroevoxine **1**—A (25 mg). Junction 12 was cleaned with methanol and the yellow crystals formed were identified as choisyine **2**—C (22 mg). Junction 15 was washed with methanol and a white precipitate was formed and identified as isobalfourodine **3** (5 mg). The quantification of the A, C and I was performed by HPLC-DAD. The HPLC system consisted of a Waters Alliance Separations module equipped with a temperature programmable auto sampler and Waters 2996 PDA detector. The LC separation was performed on a reversed phase column C18 (250 x 4.6 mm, 5μm) from Thermo Scientific, using mobile phase A (water) and mobile phase B (acetonitrile with 1% formic acid) in a gradient program with a flow of 0.8 ml/min: 0–30 min: 10% B; 30–32 min: 100% B; 32–35 min: 10% B and 35–38 min: 10% B. The volume of a single injection was 10 μl. Uv/Vis spectra between 190 and 400 nm were recorded. A seven point calibration curve was constructed by analysing various amounts (300, 250, 200, 150, 100, 50 and 25 μg/ml) of the isolated compounds (A, C and I) and four replicates were run. The calibration curves obtained for all the compounds showed a good linearity in the whole range of the tested concentrations with a regression coefficient (r2) greater than 0.96.

### Animals

Male Swiss Webster mice (18–25 g), kindly donated by Instituto Vital Brazil (Niteroi, Rio de Janeiro/Brazil), were used in this study. The animals were housed in a temperature- controlled room at 22 ± 2°C with a 12 h light/dark cycle and free access to pelleted food (Nutrilab, Brazil) and water. Twelve hours before each experiment, animals received only water in order to avoid food interference with substance absorption. Animals were acclimatized to the laboratory for at least 1 h before testing and were used only once throughout the experiments. The research was conducted in accordance with the internationally accepted guidelines for laboratory animal use and care. All procedures used followed the principles and guidelines adopted by the Brazilian College of Animal Experimentation (COBEA) and were approved by the Biomedical Science Institute/UFRJ Ethical Committee for Animal Research and received the number DFBCICB015-04/16.

### Central Antinociceptive activity—Hot Plate

Mice were treated according to the method described by Sahley and Berntson [31] and adapted by Matheus et al. [23]. Mice were placed on a hot plate (55±0.5°C) and reaction time was recorded when the animals licked one of their paws, jumped or showed an unexpected reaction at each interval of 30 minutes after oral administration via oral gavage of a solution in DMSO 1% (in saline) of 10, 30 and 100 mg/kg of EECA, 1, 3 and 10 mg/kg of A or C, or reference drug (morphine 5 mg/kg), for 180 minutes. The baseline was considered as the mean reaction time and this was obtained at 30 and 60 min before administration of the extracts, compounds or morphine. It was also defined as the normal reaction time of animal to the temperature. The increase in the baseline (%) was calculated using the equation: Increase in baseline (%) = [(reaction time x 100) / baseline]– 100.

Antinociception was quantified as area under curve (AUC) of responses from 30 to 180 min after oral drug administration. The area under the curve was calculated using GraphPad Prism (GraphPad Software Incorporated, USA).

### Evaluation of the Mechanism of Antinociceptive Action of *C*. Aztec-Pearl

The mice were pretreated with several antagonists in order to investigate the possible participation of the opioid, cholinergic, and nitric oxide systems in the antinociceptive effect of the crude ethanol extract obtained from leaves of C. Aztec-Pearl and two of the isolated alkaloids. Naloxone (1 mg/kg, i.p.), an opioid receptor antagonist, atropine (1 mg/kg, i.p.), a cholinergic receptor antagonist, yohimbine (1 mg/kg, i.p.), an adrenergic receptor antagonist, and L-nitro-arginine methyl ester (L-NAME, 3 mg/kg, i.p.), an inhibitor of nitric oxide synthase, were administered 30 minutes before 30 mg/kg, p.o., of the crude ethanol extract from leaves of C. Aztec-Pearl or 3 mg/kg of the isolated compounds. The choice of the doses of the antagonists and/or inhibitors and their treatment times were based on previous data described in the literature [32, 33] and experiments conducted in our laboratory. The dose -response curves of each antagonist were previously performed and the dose that reduced by 50% of the response of the agonist was chosen for these assays [25]. The nociceptive response was evaluated using the hot plate test.

### Acute Toxicity

Acute toxicity parameters were determined following the method described by Lorke [34]. Oral dose of the crude ethanol extract obtained from leaves of C. Aztec- Pearl (500 mg/kg) was administered to groups of ten mice (five males and five females). Parameters include convulsion, sedation, reflex, hyperactivity, increased or decreased respiration. Food and water intake were observed over a period of 5 days to analyse the behavior of the animals. After that, the stomachs of mice were removed in order to search for ulcers (single or multiple erosion, ulcer or perforation) and instances of hyperemia were counted.

### Statistical Analysis

All experimental groups for the evaluation of the anti-inflammatory and antinociceptive activities were composed of five animals. The results were presented as mean ± S.D. Statistical significance between groups was calculated by analyses of variance (ANOVA), followed by Bonferronis’s test (p < 0.05 = significant level) using GraphPad Prism (GraphPad Software Incorporated, USA).

## Results

### Phytochemical Analysis of *Choisya* Aztec-Pearl

**Fig 1:**
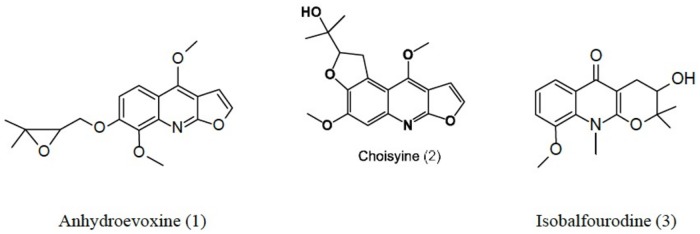
Alkaloids isolated from *C*. Aztec-Pearl.

This is the first report of the three isolated alkaloids (Anhydroevoxine **1**, Choisyine **2** and Isobalfourodine **3 –**Fig 1) in the species *C*. Aztec-Pearl. The compounds **1** and **3** are being described for the first time in *Choisya*.

A, C and isobalfourodine had their structures undoubtedly established by means of MS and NMR analysis and comparison with the literature data [11, 35, 36].

HPLC analysis allowed the quantification of the isolated alkaloids in the ethanol extract to be calculated as: Anhydroevoxyne—2.43%, Choisyine—3.71% and Isobalfourodine—1.85%.

### Central Antinociceptive activity—Hot Plate

Based on previous results by our group [14, 15, 37], we decided to evaluate the non volatile chemical composition and the possible central antinociceptive effect for *C*. Aztec- Pearl leaves, using the central model of pain (the hot plate). Because the plant and its isolated compounds showed antinociceptive action, the possible mechanism of action for such activity was investigated. For this purpose, antagonists of different pathways were used.

**Fig 2:**
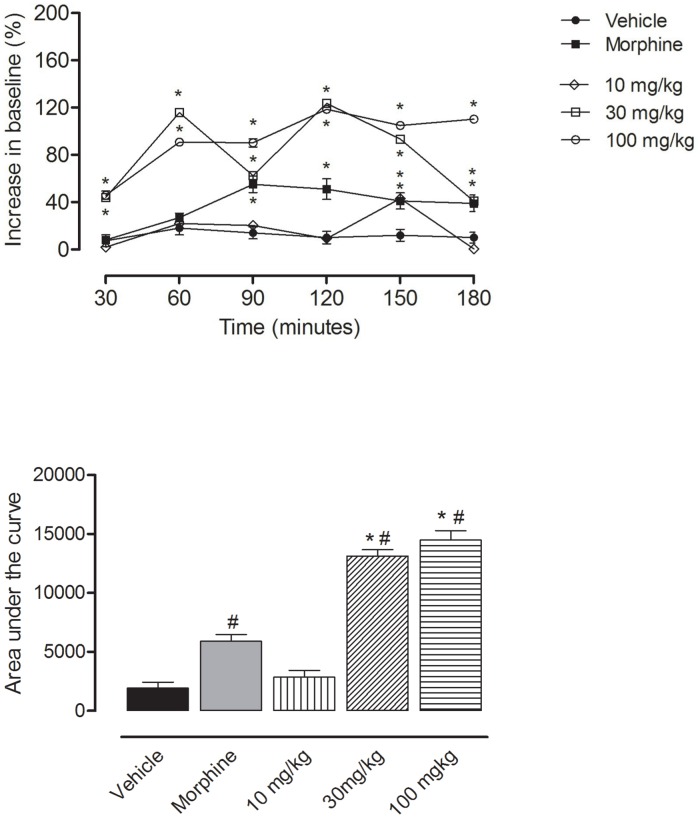
Effects of oral administration of EECA on the hot plate model. Control groups were composed either the vehicle or morphine (2.5 mg/kg, p.o.). Results are presented as mean ± S.D. (n = 6–10) of increase in baseline or area under the curve. Statistical significance was calculated using ANOVA with Bonferroni’s test where *< 0.005 when comparing the morphine, EECA-treated mice with the vehicle-treated group and #< 0.005 when comparing morphine-treated with EECA-treated mice. Where no error bars are shown, is the result of values being too small to be visible in the graphic.

Fig 2 shows the effects of EECA in the hot plate model. Pretreatment with EECA promoted a dose dependent increase in the antinociceptive response. The initial values for the action of this extract, just after 30 minutes after its administration were much higher than the ones observed for the morphine treated animals. This effect was maximum at 120 minutes following the administration of 100 mg/kg of the extract.

After this, almost no reduction in activity was observed for up to 180 minutes. Similar patterns were also observed for the other tested doses examined (30 and 10 mg/kg of this extract). At 30 mg/kg the maximum activity was also observed at 120 minutes and the decrease was observed reaching values similar to those in the morphine-treated group at 180 minutes. The treated group at 10 mg/kg showed a slight increase in the baseline, always smaller than the one observed for the morphine treated group. The exception being the value reached at 150 minutes, which was similar to the one seen for morphine. All doses which tested significantly cause an increase in the antinociceptive activity when compared with the vehicle group and this effect was higher than that observed for morphine (at 30 and 100 mg/kg doses). The analgesic effect of morphine was observed 60 min after administration and reached a plateau between 90 and 150 minutes. The values obtained for morphine were converted to a graph of area under the curve, and a value of 5,193 was obtained for this agonist. The vehicle did not show antinociceptive response in the hot plate test.

Isolated compounds C and A were also tested in the hot plate model. For the isolated compounds doses of 1, 3 and 10 mg/kg were used. The isolated compounds behaved in a similar way to the extract with their higher doses of 3 and 10 mg/kg providing a higher effect than morphine while the doses of 1 mg/kg provided similar results to morphine treated animals.

**Fig 3:**
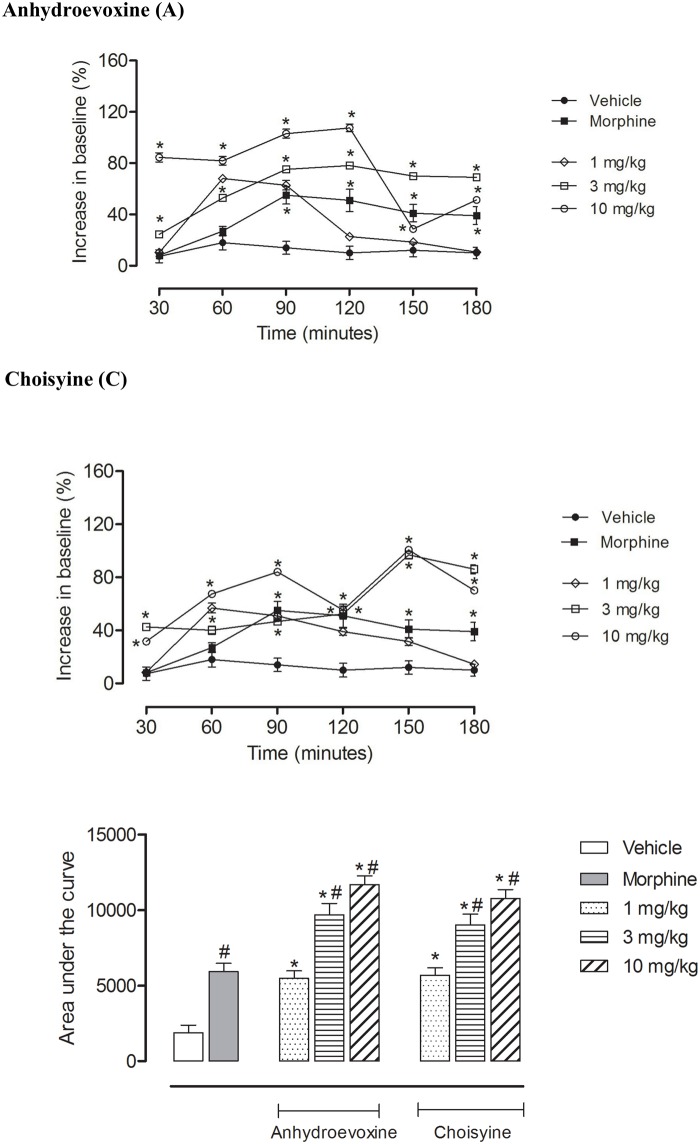
Effects of oral administration of A or C on the hot plate model. Control groups were composed of vehicle or morphine (2.5 mg/kg, p.o.). Results are presented as mean ± S.D. (n = 6–10) of increase in baseline or area under the curve. Statistical significance was calculated using ANOVA with Bonferroni’s test where * < 0.005 when comparing the morphine, A or C-treated mice with the vehicle-treated group and # < 0.005 when the C- and A-treated mice were compared with the morphine-treated mice. Where no error bars are shown, is the result of values being too small to be visible in the graphic.

Anhydroevoxine (at 3 and 10 mg/kg) significantly increased the reaction time in the mice. The maximum effect for this substance was achieved at 120 minutes, with a decrease below the morphine levels at 150 minutes and an increase back to a higher value again at 180 minutes (Fig 3).

Choisyine (at 3 and 10 mg/kg) significantly increased the reaction time in the mice. The maximum effect for this substance was achieved at 150 minutes, with a slight decay at 180 minutes (Fig 3).

The graph showing the area under the curve (Figs 2 and 3) demonstrate that EECA at 30 and 100 mg/kg and A and C at 3 and 10 mg/kg display a much higher antinociceptive activity than the one observed for morphine.

### Mechanism of Action of *C*. Aztec-Pearl and its alkaloids

**Fig 4:**
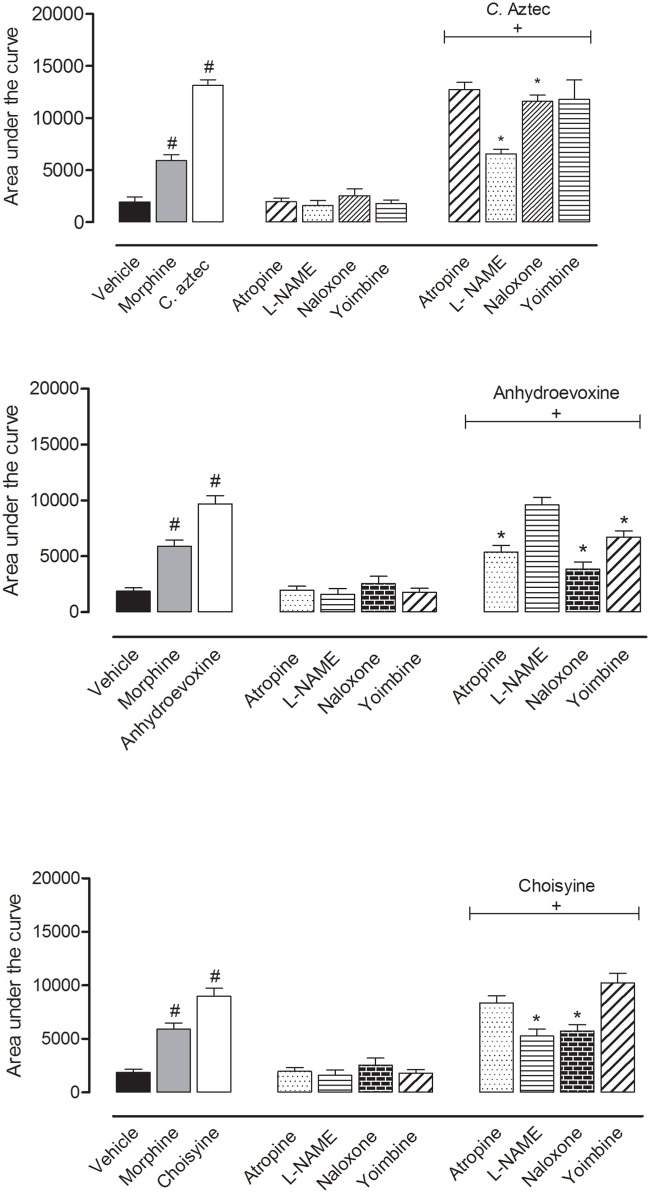
Effects of different antagonists on antinociceptive activity of EECA, A and C in the hot plate model. Animals were pretreated with atropine (1 mg/kg, i.p.), L-NAME (3 mg/kg, i.p.), Yohimbine (1 mg/kg, i.p.) or naloxone (1 mg/kg, i.p.) 30 minutes prior to oral administration of EECA (30 mg/kg) and A or C (3 mg/kg). The results are presented as mean ± S.D. (n = 6–10) of area under the curve calculated using Prism Software 5.0. Statistical significance was calculated using ANOVA followed by Bonferroni’s test. ** < 0.005 when comparing the morphine- or the EECA, A or C-treated mice with vehicle-treated.

As all doses of EECA as well as A and C showed a significant effect in the hot plate model, we decided to investigate the possible mechanism by which the antinociceptive activity occurs. The results indicate that naloxone and L-Name reverted the antinociceptive effect observed for EECA in a significant way. Choisyine behaved in a similar fashion when compared to the crude extract, showing involvement of NO and opiate pathways. Anhydroevoxine also shows the involvement of the opiate pathway but we could also see that the adrenergic pathway plays a role, with the reversal of the antinociceptive effect when Yohimbine was used (Fig 4).

### Assessment of Side Effects and Toxicity

A single oral administration of EECA (at 100mg/kg) did not induce significant mucosal lesion 4 hours after their administration, presenting visual conditions similar to those for the vehicle. EECA did not alter the fall latency or number of falls in the rotarod test or the number of crossings or rearing responses when compared with the saline group. Similar pattern was also checked for the two alkaloids at 10 mg/kg (data not shown).

## Discussion

In the present study it has been shown that EECA, A and C displayed a pronounced central antinociceptive effect. The mechanism of their action by which extract and compounds exert their action was also investigated. The hot plate test has been used by many investigators and has been found to be suitable for the evaluation of centrally but not peripherically acting analgesics [38–40]. Characteristic differences occurred in the time course and maximal effects of the antinociceptive action of EECA, A and C when comparing with morphine-treated group. A rapid onset with an early maximum effect is characteristic of the time course of action of opioid agonists (e.g., morphine), which mediate analgesia via opioid receptors under both normal and inflammatory conditions [41]. The administration of EECA, A and C produced a time course of action much higher when compared to morphine. One possible explanation for this rapid onset of action might be the solubility of the substances, which allows them to rapidly reach the brain. Pain sensation can be effectively controlled by systems of neurotransmitters such as oxidonitrergic, opioid, adrenergic and cholinergic systems, in addition to others, acting in different ways during the pain transmission process and the interference with one of the components makes them a very interesting and complex phenomenon [42]. To study whether EECA, A and C develop their analgesic properties interfering with one of these components we evaluated their mechanism of action by pretreating the animals with some inhibitors of the systems listed above. Among the systems involved in pain, the opioid system is one of the most important. This system participates in both the perception and modulation of the pain process by both central and peripheral mechanisms [43]. To evaluate the participation of the opioid system, mice were pretreated with naloxone, an opioid antagonist. The results obtained clearly demonstrated that the opioid system is intensely responsible for the antinociceptive activity of this extract. This observation is clearly illustrated by the fact that naloxone reverts the analgesic effect observed for EECA. These results allowed us to suggest that EECA contains several substances with significant effects on opioid receptors in the central nervous system. In fact similar profiles were found when mice were pretreated with naloxone and A or C. They were also acting via the opiate receptor. Several studies have also demonstrated that cholinergic pathways are implicated in antinociception at the spinal level [44–47]. It is known that the atropine-induced cholinergic receptor blockade is an important effect that involves the medullar and supramedullar centres related to antinociception [48]. Moreover, cholinergic pain modulation may also involve first-order neurons (sensory neurons responsible for delivering sensory information to the CNS) via muscarinic acetylcholine M2 receptors. In this context, Dussor and colleagues (2004) [49] have demonstrated that activating peripheral M2 receptors can lead to antinociception *in vivo* and inhibit antinociceptive activity in vitro. In our study, we also demonstrated that systemic atropine did not influence the antinociceptive effect of EECA or A. Only for C we observed a modulation through this pathway.

The oxidonitrergic pathway is also important for the action of this extract. To evaluate the participation of the oxidonitrergic system, mice were pretreated with L-Name, an antagonist of this system. Our data clearly demonstrated that the oxidonitrergic system is also responsible for the antinociceptive activity of EECA. This observation is clearly illustrated by the fact that L-Name reverts the analgesic effect observed for this extract. The same is observed for the isolated quinoline alkaloid A.

Although the doses of EECA used were higher than traditional analgesic drugs, one must take into account the fact that EECA is not a pure drug or synthetic compound. It has different constituents at different concentrations. Another fact is that EECA was administered orally. The absorption by gastrointestinal tract could be influenced by the pH in the stomach and the liposolubility of its constituents may interfere with the absorption. Our results were also able to clarify the mechanism of the action of the crude ethanol extract obtained from leaves of C. Aztec-Pearl. We also proved the very low toxic profile of this plant extract on animals, similar results were shown for A and C, compounds isolated from this plant.

## Conclusion

In summary, results point out to an incredible antinociceptive activity for EEAC that is also observed for A and C and this can open a window for further investigations of this type of alkaloids and other constituents/other extracts obtained from plants of this genus in relation to this very important pharmacological activity.
